#  O apoio às cuidadoras familiares de pessoas idosas no contexto da pandemia de COVID-19 

**DOI:** 10.1590/0102-311XPT072423

**Published:** 2023-12-15

**Authors:** Dalia Elena Romero, Daniel Groisman, Leo Ramos Maia

**Affiliations:** 1 Instituto de Comunicação e Informação Científica e Tecnológica em Saúde, Fundação Oswaldo Cruz, Rio de Janeiro, Brasil.; 2 Escola Politécnica de Saúde Joaquim Venâncio, Fundação Oswaldo Cruz, Rio de Janeiro, Brasil.; 3 Escola Nacional de Saúde Pública Sergio Arouca, Fundação Oswaldo Cruz, Rio de Janeiro, Brasil.

**Keywords:** Cuidadores, Família, Idoso, Apoio Social, Caregivers, Family, Aged, Social Support, Cuidadores, Familia, Anciano, Apoyo Social

## Abstract

Este estudo objetiva identificar o tipo de apoio recebido por cuidadoras familiares de pessoas idosas na pandemia de COVID-19, assim como os fatores socioeconômicos de saúde e relativos ao cuidado associados. Utilizou-se dados do estudo *CUIDA-COVID*, questionário *online* realizado entre agosto e novembro de 2020 (n = 4.820). Foram construídos três modelos múltiplos, tendo como desfecho: apoio contratado, apoio familiar e ausência de apoio. As variáveis independentes relacionadas a aspectos socioeconômicos, de saúde e cuidado foram convertidas em categorias dicotômicas. O apoio familiar foi o mais comum (48,6%), seguido pelo ausência de apoio (38,3%) e apoio contratado (13,1%). Não foram observadas associações significativas entre raça/cor, situação de pobreza, problema crônico de coluna e estado de ânimo negativo com nenhum dos tipos de apoio. Foram fatores fortemente associados (RPajustada > 2,0) ao apoio contratado: a não diminuição acentuada da renda na pandemia; a não dedicação ao cuidado em tempo integral; a autoavaliação da saúde boa/excelente; e o alto grau de dependência da pessoa idosa. Para o apoio familiar, as principais associações foram: o não aumento acentuado do esforço dedicado ao cuidado na pandemia; a não dedicação ao cuidado em tempo integral; e a diminuição acentuada da renda na pandemia. Foram as características associadas ao ausência de apoio: viver com o idoso; dedicar-se ao cuidado em tempo integral; residir em domicílios com uma ou duas pessoas; e relatar um aumento acentuado do esforço dedicado ao cuidado na pandemia. No geral, as cuidadoras com apoio contratado tiveram as melhores condições socioeconômicas, de cuidado e saúde. A desassistência foi relacionada a piores condições de saúde e cuidado, enquanto o apoio familiar às piores condições socioeconômicas.

## Abstract (en)

This study aims to identify the type of support received by family caregivers of older people during the COVID-19 pandemic, as well as the associated socioeconomic health and care-related factors. Data from *CUIDA-COVID* study, an online questionnaire conducted from august to november 2020 (n = 4,820) were used. Three multiple models were built, with the following outcome: contracted support, family support, and lack of support. Independent variables related to socioeconomic, health, and care aspects were converted into dichotomous categories. Family support was the most common (48.6%), followed by lack of support (38.3%), and contracted support (13.1%). No significant associations were observed between race/skin color, poverty status, chronic back pain and negative mood with any type of support. Factors strongly associated (adjustedPR > 2.0) with contracted support were: the non-sharp decrease in income during the pandemic; non-dedication to full-time care; self-rated good/excellent health; and the high degree of dependence of the older individual. For family support, the main associations were: the non-marked increase in the effort to care during the pandemic, the non-dedication to full-time care and the sharp decrease in income during the pandemic. The characteristics associated with lack of support were: living with the older person; dedication to full-time care; living in households with 1 or 2 people; and report a sharp increase in effort to care during the pandemic. Overall, caregivers with contracted support had the best socioeconomic, care and health conditions. Lack of support was related to worse health and care conditions, while family support was related to worse socioeconomic conditions.

## Resumen

Este estudio tiene por objetivo identificar el tipo de apoyo recibido por las cuidadoras familiares de personas mayores en la pandemia de la COVID-19 y sus factores socioeconómicos de salud y atención asociados. Se utilizaron los datos del estudio *CUIDA-COVID*, un cuestionario en línea aplicado entre agosto y noviembre de 2020 (n = 4.820). Se construyeron tres modelos múltiples, teniendo en cuenta el resultado: apoyo contratado, apoyo familiar y falta de apoyo. Las variables independientes relacionadas con los aspectos socioeconómicos, de salud y de atención se convirtieron en categorías dicotómicas. El apoyo familia fue el más frecuente (48,6%), seguido de la falta de apoyo (38,3%) y el apoyo contratado (13,1%). No se observaron asociaciones significativas entre raza/color, pobreza, problemas crónicos de espalda y estado de ánimo negativo con ninguno de los tipos de apoyo. Los siguientes factores se asociaron fuertemente (RPajustado > 2,0) con el apoyo contratado: la falta de descenso brusco de los ingresos en la pandemia, la falta de dedicación a la atención a tiempo completo, la autoevaluación de la salud como buena/excelente, y el alto grado de dependencia de las personas mayores. Las principales asociaciones en el apoyo familia fueron las siguientes: ascenso no brusco del esfuerzo dedicado a la atención en la pandemia, la no dedicación a una atención a tiempo completo y la fuerte caída de los ingresos en la pandemia. Las características asociadas a la falta de apoyo fueron las siguientes: vivir con la persona mayor, dedicarse a una atención a tiempo completo, vivir con 1 o 2 personas en el hogar, y reportar un marcado incremento en el esfuerzo dedicado a la atención en la pandemia. En general, las cuidadoras con apoyo contratado tenían las mejores condiciones socioeconómicas, de atención y de salud. La falta de atención se relacionó con peores condiciones de salud y atención, mientras que el apoyo familia se relacionó con peores condiciones socioeconómicas.

## Introdução

Cuidar não é uma responsabilidade nova para a família. O papel de seus membros como fonte de apoio emocional, físico e financeiro é de longa data ^1^. Entretanto, o aumento da longevidade de pessoas idosas com doenças crônicas e/ou limitações funcionais, sem uma adequada adaptação dos sistemas de saúde, levou a importantes mudanças no âmbito familiar nas últimas décadas. O número de cuidadores familiares aumentou, assim como a duração, a intensidade e a complexidade do cuidado prestado ^2^.

No Brasil, a cultura impõe uma carga excessiva de responsabilidade aos familiares ^3^. Segundo o *Estudo Longitudinal de Saúde dos Idosos Brasileiros* (ELSI-Brasil) de 2015-2016, 94% das pessoas idosas que dependem de cuidados têm como principal cuidador um familiar ^4^. Isso configura um problema porque, no geral, assumir as funções do cuidado no âmbito familiar é uma experiência que gera estresse e sobrecarga emocional e física, conforme demonstrado em diversos estudos ^5^^,^^6^^,^^7^.

O apoio social - compreendido como o fornecimento por outras pessoas de recursos emocionais, como demonstrações afeto; instrumentais, que envolvem o auxílio de necessidades materiais; informacionais, que podem ajudar na resolubilidade dos problemas; e interacionais positivos, que se referem à disponibilidade de acesso a pessoas que proporcionem momentos de descontração - tem sido fortemente relacionado à qualidade de vida de cuidadores familiares ^8^. A percepção do apoio recebido e a relação do cuidador com o provedor do apoio são fatores que influenciam nos seus benefícios ^9^.

Nesse sentido, a presença de terceiros para compartilhar responsabilidades reduz a exposição dos cuidadores familiares à sobrecarga derivada do cuidado ^8^^,^^10^^,^^11^^,^^12^. Entretanto, se por um lado o apoio de cuidadores formais (remunerados) ainda é restrito devido ao alto custo ^13^, por outro, há uma tendência de redução do potencial assistencial informal (familiar), devido à diminuição do tamanho e diversidade dos arranjos familiares, bem como à mudança do papel da mulher na sociedade e à incipiente participação masculina na prestação de cuidados ^14^. No contexto da pandemia de COVID-19, a necessidade de distanciamento social restringiu ainda mais a disponibilidade de terceiros para o apoio ao cuidado de pessoas idosas ^15^.

A formação histórica e cultural do cuidar, conformada sob relações fortemente hierárquicas, tornou-o uma função subvalorizada e invisibilizada ^16^^,^^17^. Isso explica por que mulheres ^4^^,^^7^, especialmente negras ^16^, são as principais designadas para o cuidado no ambiente doméstico.

Estudos voltados à população de cuidadores familiares têm evidenciado os efeitos negativos do cuidar com maior frequência do que os positivos ^1^. Problemas de saúde física, como problema crônico de coluna, varizes e hipertensão ^5^, e mental, como sono reduzido e sintomas depressivos e ansiosos ^18^, têm se mostrado mais prevalentes nesse grupo, quando comparado à população em geral. Desvantagens econômicas de cuidadores familiares também foram relatadas ^19^^,^^20^.

Estudos de abrangência nacional que considerem a situação dos cuidadores familiares de pessoas idosas são escassos ^4^^,^^21^. Em sua especificidade, a identificação da indisponibilidade ou do tipo do provedor do apoio social, em termos de Brasil, é tema que carece de análises na literatura ^9^, sobretudo durante a pandemia, período em que as interações sociais foram dificultadas.

Diante disso, este estudo tem o objetivo de identificar o tipo de apoio recebido pelas cuidadoras familiares de pessoas idosas na pandemia de COVID-19 no Brasil, bem como os fatores socioeconômicos e relativos às condições de saúde e atividades de cuidado associados.

## Métodos

### Fonte de dados e amostra

No Brasil, entre agosto e novembro de 2020, foi realizado o estudo *CUIDA-COVID: Pesquisa Nacional sobre as Condições de Trabalho e Saúde das Pessoas Cuidadoras de Idosos na Pandemia*^22^ com a finalidade de descrever as mudanças na vida dos cuidadores de pessoas idosas durante a pandemia de COVID-19. Uma das seções da pesquisa foi direcionada a cuidadores familiares. Este estudo se baseia nos dados dessa pesquisa.

Realizada por meio de questionários *online*, a pesquisa foi elaborada pela Fundação Oswaldo Cruz (Fiocruz) e aprovada pelo Comitê de Ética em Pesquisa da Escola Politécnica de Saúde Joaquim Venâncio (EPSJV/Fiocruz; CAAE 33616320.8.0000.5241). Todas as informações coletadas foram anônimas, preenchidas pelo próprio indivíduo e hospedadas em *website* nos servidores do Instituto de Comunicação e Informação Científica e Tecnológica em Saúde (ICICT/Fiocruz).

O inquérito utilizou várias estratégias de divulgação para alcançar o público-alvo, incluindo a criação de um *website* para o projeto, divulgação por meio dos setores de jornalismo da Fiocruz e a elaboração de materiais audiovisuais para compartilhamento em redes sociais. Além disso, foram contatados gestores, lideranças comunitárias e outros atores do Estado e da sociedade civil para colaboração na divulgação do estudo. Maiores detalhes da CUIDA-COVID estão no relatório da pesquisa ^22^.

A amostra final da pesquisa foi composta por 4.820 cuidadores de pessoas idosas, sendo 2.354 remunerados e 2.466 não remunerados. Para este estudo, foram selecionadas apenas as cuidadoras não remuneradas, mulheres e que responderam à pergunta “Além de você, outra pessoa se responsabiliza pelo cuidado da pessoa idosa?”, totalizando uma amostra de 2.023.

A pergunta “De quem você cuida, de forma não remunerada?” identificou o tipo de vínculo com a pessoa cuidada por meio de três alternativas: (1) familiar ou parente; (2) amigo(a) ou vizinho(a); e (3) outro. Como a maioria das entrevistadas (86,3%) escolheu a primeira opção, neste estudo optou-se por utilizar o termo “cuidadoras familiares” para se referir à população analisada. Para ajustar a amostra de acordo com as condições socioeconômicas, foram realizadas ponderações calculadas por procedimentos de pós-estratificação ^23^, levando em consideração o grau de escolaridade da população de cuidadores familiares brasileiros, identificado na *Pesquisa Nacional por Amostra por Domicílios* (PNAD) de 2019.

### Variáveis

Para identificar o tipo de apoio recebido pelas cuidadoras, utilizou-se a pergunta “Além de você, outra pessoa se responsabiliza pelo cuidado da pessoa idosa?”. As opções de resposta eram: (1) sim, um(a) cuidador(a) contratado(a); (2) sim, outro familiar, parente, vizinho, amigo; (3) não, sou o(a) único(a) cuidador(a) responsável. Dessa forma, foram definidos três grupos de análise: apoio contratado, apoio familiar e ausência de apoio, respectivamente.

As características socioeconômicas das cuidadoras familiares de pessoas idosas analisadas incluíram a raça/cor da pele, situação de pobreza monetária, situação de trabalho antes da pandemia e número de moradores do domicílio. A raça/cor da pele foi categorizada em branca ou negra, essa última composta por pretos e pardos. Cuidadoras que responderam às demais categorias (amarelo ou indígena) corresponderam a 2,8% da amostra e não foram incorporadas à análise dessa variável específica.

A situação de pobreza monetária foi identificada a partir da renda *per capita* domiciliar em salários mínimos, obtida pela pergunta: Qual é atualmente a sua renda mensal familiar? A resposta foi dividida pelo total de membros do domicílio e classificada nas faixas: até ½ salário mínimo e mais de ½ salário mínimo. As primeiras foram consideradas como em situação de pobreza e as últimas como não pobres. O ponto de corte da linha de pobreza foi definido de acordo com o limite de renda para cadastramento no Cadastro Único de Programas Sociais do Governo Federal (CadÚnico) ^24^.

A situação do trabalho antes da pandemia foi obtida pela pergunta “Antes do início da pandemia do coronavírus, qual era a sua situação de trabalho?”. Aquelas que responderam que trabalhavam com ou sem carteira assinada foram categorizadas em “trabalhava antes da pandemia”. As demais respostas foram categorizadas como “não trabalhavam antes da pandemia”.

O número de moradores do domicílio foi obtido por meio da pergunta “No seu domicílio, qual é o número de moradores?” e categorizado em “1 ou 2” e “3 ou mais”.

O grau de dependência e o tempo que cuida da pessoa idosa foram agrupados como características do cuidado independentes da pandemia. A primeira foi obtida a partir da pergunta “Que atividades você costuma realizar no seu trabalho de cuidados?”, aquelas que declararam auxiliar no banho e/ou na alimentação foram consideradas cuidadoras de pessoas idosas com alta dependência ^25^. A segunda foi obtida a partir da pergunta “Há quanto tempo você cuida deste(a) idoso(a)?” e categorizada em “até 3 anos” e “mais de 3 anos”.

Também foram analisadas variáveis relativas às condições de saúde, cuidado e renda atreladas à pandemia. As variáveis do primeiro grupo foram: (1) a autoavaliação da saúde, obtida a partir da pergunta “Em geral, como você avalia sua saúde?” e categorizada em “moderada/ruim/péssima” e “boa/excelente”; (2) o problema crônico de coluna; identificado a partir da pergunta “Você tem algum problema crônico de coluna, como dor crônica nas costas ou no pescoço, lombalgia, dor ciática, problemas nas vértebras ou disco?” (sim ou não); (3) o estado de ânimo muito ruim, identificado a partir das respostas “muitas vezes” ou “sempre”, tanto à pergunta “No período da pandemia, com que frequência você se sentiu triste ou deprimido(a)?”, quanto à pergunta “No período da pandemia, com que frequência você se sentiu ansioso(a) ou nervoso(a)?”.

Cinco variáveis relativas às condições do cuidado na pandemia foram analisadas:

(I) A dedicação ao cuidado em tempo integral, identificada a partir das perguntas “Quantos dias da semana você trabalha como cuidador(a) em média?”, “Em um dia típico, quantas horas você trabalha como cuidador(a)?”. Foram consideradas cuidadoras dedicadas ao cuidado em tempo integral aquelas que disseram trabalhar como cuidadora 24 horas, todos os dias na semana;

(II) residir com a pessoa idosa, obtida a partir da pergunta “Você mora na mesma casa ou terreno que a pessoa cuidada?” (sim ou não);

(III) a realização de atividades domésticas entre as tarefas do cuidado, obtida a partir da seleção de pelo menos uma das opções: “preparação de alimentos”, “limpeza do domicílio” e “lavar e/ou passar roupas”, à pergunta “Que atividades você costuma realizar no seu trabalho de cuidados?”;

(IV) o aumento acentuado do tempo de dedicação ao cuidado na pandemia, obtido a partir da pergunta “Seu tempo de dedicação para os cuidados mudou depois do início da pandemia?”, categorizado em “aumentou muito” ou “não aumentou muito”;

(V) o aumento acentuado do esforço dedicado ao cuidado na pandemia, obtido a partir da pergunta “A quantidade de esforço que você precisa dedicar para o trabalho de cuidados se alterou depois do início da pandemia?”, categorizado em “aumentou muito” ou “não aumentou muito”.

A diminuição acentuada da renda familiar na pandemia foi obtida a partir da pergunta “Sua renda familiar se alterou após o início da pandemia?”. Aquelas que selecionaram a alternativa “diminuiu muito” foram consideradas como tendo uma diminuição acentuada da renda.

### Análise

Foi analisada a distribuição percentual dos grupos de variáveis mencionadas, com base no tipo de apoio recebido. Calculou-se a prevalência das cuidadoras familiares considerando diferentes tipos de apoio: contratado, familiar e ausência de apoio. Fatores socioeconômicos, de saúde, cuidado e sua relação com a pandemia foram considerados como variáveis independentes. Foram criados modelos de razão de prevalência (RP) simples para examinar os fatores associados a cada tipo de apoio. Três modelos múltiplos foram desenvolvidos para cada tipo de apoio, usando variáveis *dummy* correspondentes.

Intervalos de 95% de confiança (IC95%) foram calculados para todas as estimativas, e o teste qui-quadrado de Pearson foi realizado. A análise foi conduzida por meio da regressão de Poisson, usando uma abordagem de variância robusta. Variáveis com valor de p < 0,05 foram consideradas estatisticamente significativas. A idade foi considerada um fator de ajuste em todos os modelos. Todas as variáveis, mesmo as não significativas no modelo bivariado, foram incluídas na análise multivariada para obter medidas de efeito independentes dos fatores analisados.

## Resultados

O perfil socioeconômico precário das cuidadoras familiares de pessoas idosas que atuavam na pandemia é verificado na Tabela 1. Entre o total de cuidadoras, a maioria era pobre (56,2%), não tinha trabalho remunerado antes da pandemia (61%) e morava em domicílios com três ou mais pessoas (74,3%). A distribuição racial entre cuidadoras negras e brancas demonstrou-se semelhante.

**Tabela 1:** Distribuição percentual das características socioeconômicas, de saúde e relacionados ao cuidado, independentes e atreladas à pandemia entre cuidadoras familiares de pessoas idosas, segundo o tipo de apoio recebido.

Variáveis	Total			Apoio contratado			Apoio familiar			Ausência de apoio			Valor de p
	n	%	IC95%	n	%	IC95%	n	%	IC95%	n	%	IC95%	
Total	2.023	100,0	-	264	100,0	-	984	100,0	-	775	100,0	-	-
Características socioeconômicas e do cuidado independentes da pandemia													
Raça/Cor da pele													
Branca	1.022	52,0	49,8-54,2	145	59,6	53,4-65,8	466	48,2	45,0-51,3	411	54,7	51,1-58,2	0,001
Negra	941	48,0	45,8-50,2	99	40,4	34,2-46,6	502	51,8	48,7-55,0	340	45,3	41,8-48,9	
Situação de pobreza monetária													
Não pobre	756	43,8	40,3-47,4	123	56,6	53,0-60,1	328	38,7	35,2-42,1	305	46,0	42,4-49,5	< 0,001
Pobre	973	56,2	53,8-58,5	95	43,4	36,9-50,0	519	61,3	58,1-64,6	359	54,0	50,2-57,8	
Situação do trabalho antes da pandemia													
Trabalhava	798	39,0	36,9-41,2	130	50,8	44,8-56,9	563	42,7	39,6-45,8	531	31,5	28,2-34,7	< 0,001
Não trabalhava	1.224	61,0	58,8-63,1	134	49,2	43,1-55,2	420	57,3	54,2-60,4	244	68,5	65,3-71,8	
Número de moradores no domicílio													
1 ou 2	490	25,7	23,7-27,6	77	32,3	26,4-38,2	202	21,1	18,5-23,7	211	28,4	25,1-31,6	< 0,001
3 ou mais	1.449	74,3	72,1-76,6	162	67,7	65,3-70,1	754	78,9	76,8-81,0	533	71,6	69,3-73,9	
Alto grau de dependência													
Não	1.028	50,8	48,6-53,0	94	35,6	29,8-41,4	530	53,9	50,8-57,0	404	52,1	48,6-55,6	< 0,001
Sim	994	49,2	47,0-51,4	170	64,4	58,6-70,2	453	46,1	43,0-49,2	371	47,9	44,4-51,4	
Tempo que cuida da pessoa idosa (anos)													
Até 3	765	38,0	34,6-41,5	106	40,0	36,5-43,4	425	43,5	40,0-47,0	234	30,3	27,0-33,5	< 0,001
Mais de 3	1.249	62,0	59,8-64,1	158	60,0	54,1-65,9	553	56,5	53,4-59,6	538	69,7	66,5-73,0	
Condições de saúde, cuidado e renda atreladas à pandemia													
Autoavaliação da saúde moderada/ruim/péssima	850	42,4	40,2-44,5	69	26,5	21,2-31,9	393	40,0	36,9-43,0	388	51,1	47,5-54,6	< 0,001
Problema crônico de coluna	956	47,7	45,5-49,9	112	42,4	36,4-48,4	437	45,0	41,9-48,1	407	52,7	49,2-56,2	0,001
Estado de ânimo muito ruim	886	43,9	41,7-46,1	96	36,7	30,8-42,5	393	40,1	37,0-43,2	397	51,4	47,9-54,9	< 0,001
Dedicação ao cuidado em tempo integral	766	38,1	36,0-40,3	32	12,1	8,2-16,0	270	27,7	24,8-30,5	464	60,0	56,6-63,5	< 0,001
Reside com a pessoa idosa	1.327	65,2	63,1-67,3	111	42,0	36,0-47,9	553	56,4	53,3-59,5	663	85,6	83,1-88,0	< 0,001
Realiza atividades domésticas entre as tarefas do cuidado	1.722	83,7	82,1-85,3	200	75,6	70,4-80,7	801	81,5	79,0-83,9	721	93,1	91,3-94,9	< 0,001
Aumento acentuado do tempo dedicado ao cuidado	865	56,7	54,5-58,9	123	53,3	47,2-59,3	379	61,3	58,3-64,4	363	53,2	49,7-56,7	0,001
Aumento acentuado do esforço dedicado ao cuidado	779	39,1	36,9-41,2	108	40,9	35,0-46,8	327	33,3	30,3-36,2	344	44,4	40,9-47,9	< 0,001
Diminuição acentuada da renda familiar	215	10,8	9,4-12,1	18	6,9	3,8-9,9	126	12,9	10,8-15,0	71	9,2	7,2-11,3	0,005

IC95%: intervalo de 95% de confiança.

Fonte: Groisman et al. ^22^.

Cerca de 60% atuavam há mais de três anos e metade prestava assistência a pessoas idosas com alto grau de dependência. Ao redor de 45% das cuidadoras relataram autoavaliação de saúde moderada/ruim/péssima, problemas crônicos de coluna e estado de ânimo muito ruim. Quase 40% se dedicavam ao cuidado em tempo integral e relataram um aumento acentuado do esforço dedicado ao trabalho de cuidar. Quatro em cada cinco cuidadoras exerciam atividades domésticas entre as tarefas relacionadas ao cuidado e a maioria residia com a pessoa idosa cuidada. De cada dez, uma teve a renda familiar muito diminuída durante a pandemia.

Observa-se que aquelas que contavam com apoio familiar apresentavam desvantagens socioeconômicas em comparação às demais. Tal realidade se agravou com a pandemia, tendo em vista que o relato de diminuição acentuada da renda familiar foi mais frequente entre elas. Por sua vez, as cuidadoras com ausência de apoio apresentaram piores condições de saúde e nas atividades do cuidado durante a pandemia. As maiores desvantagens são verificadas na dedicação ao cuidado em tempo integral, na autoavaliação da saúde, no tempo de atuação como cuidadora e no estado de ânimo muito ruim. Já as cuidadoras com apoio contratado, apesar de mais frequentemente cuidarem de pessoas idosas com alto grau de dependência, apresentaram melhores condições socioeconômicas, de saúde e do cuidado, tanto nas variáveis atreladas à pandemia quanto nas independentes.

Cuidadores contratados apoiavam 13,1% (IC95%: 11,6-14,6) das cuidadoras (Tabela 2). Essa proporção ultrapassava os 20% entre cuidadoras que não moravam com a pessoa idosa cuidada e que não realizavam atividades domésticas como tarefas de cuidado e chegava aos 17,1% (IC95%: 14,8-19,4).

**Tabela 2:** Prevalência e razões de prevalência (RP), bruta e ajustada, das características socioeconômicas e de saúde relacionadas ao cuidado de cuidadores familiares que recebem apoio contratado. Brasil, 2020 (n = 2.023).

Variáveis	%	IC95%	RP	IC95%	Valor de p	RPajustada *	IC95%	Valor de p
Total	13,1	11,6-14,6	-	-	-	-	-	-
Características socioeconômicas e do cuidado independentes da pandemia								
Raça/Cor da pele								
Branca	14,2	12,1-16,3	1,15	0,86-1,52	0,347	1,11	0,77-1,59	0,575
Negra	10,5	8,5-12,5	1,00	-		1,00	-	
Situação de pobreza monetária								
Não pobre	16,3	13,7-18,9	1,57	1,12-2,20	0,008	1,07	0,76-1,52	0,694
Pobre	9,8	7,9-11,7	1,00	-		1,00	-	
Situação do trabalho antes da pandemia								
Trabalhava	16,8	14,2-19,4	1,83	1,4-2,38	< 0,001	1,26	0,89-1,78	0,194
Não trabalhava	10,6	8,9-12,9	1,00	-		1,00	-	
Número de moradores no domicílio								
1 ou 2	15,8	12,6-19,0	1,27	0,93-1,74	0,130	0,68	0,49-0,95	
3 ou mais	11,2	9,6-12,8	1,00	-		1,00	-	0,023
Alto grau de dependência								
Não	9,1	7,3-10,9	1,00	-	< 0,001	1,00	-	< 0,001
Sim	17,1	14,8-19,4	2,01	1,51-2,66		2,14	1,47-3,13	
Tempo que cuida da pessoa idosa (anos)								
Até 3	13,8	11,4-16,2	1,15	0,88-1,50	0,316	1,00	0,73-1,35	0,979
Mais de 3	12,7	10,9-14,5	1,00	-		1,00	-	
Condições de saúde, cuidado e renda atreladas à pandemia								
Autoavaliação da saúde								
Boa/Excelente	16,5	14,4-18,6	2,19	1,59-3,02	< 0,001	2,83	1,58-5,05	< 0,001
Moderada/Ruim/Péssima	8,1	6,3-9,9	1,00	-		1,00	-	
Problema crônico de coluna								
Não	14,4	12,3-16,5	1,42	1,06-1,90	0,020	1,41	0,91-2,18	0,126
Sim	11,7	9,7-13,7	1,00	-		1,00	-	
Estado de ânimo muito ruim								
Não	14,7	12,6-16,8	1,47	1,11-1,95	0,008	0,93	0,60-1,44	0,730
Sim	10,9	8,8-13,0	1,00	-		1,00	-	
Dedicação ao cuidado em tempo integral								
Não	18,6	16,4-20,8	5,78	3,64-9,16	< 0,001	3,02	1,62-5,64	< 0,001
Sim	4,2	2,8-5,6	1,00	-		1,00	-	
Reside com a pessoa idosa								
Não	22,1	19,0-25,2	3,69	2,78-4,89	< 0,001	1,95	1,31-2,90	0,001
Sim	8,4	6,9-9,9	1,00	-		1,00	-	
Realiza atividades domésticas entre as tarefas do cuidado								
Não	21,5	16,9-26,1	1,82	1,34-2,47	< 0,001	1,80	1,24-2,59	0,002
Sim	11,6	10,1-13,1	1,00	-		1,00	-	
Aumento acentuado do tempo dedicado ao cuidado								
Não	12,2	10,3-14,1	1,00	-	0,122	1,00	-	0,287
Sim	14,3	12,0-16,6	1,23	0,95-1,59		0,75	0,44-1,27	
Aumento acentuado do esforço dedicado ao cuidado								
Não	12,6	10,8-14,4	1,00	-	0,430	1,00	-	0,029
Sim	13,9	11,5-16,3	1,12	0,85-1,48		1,86	1,06-3,26	
Diminuição acentuada da renda familiar								
Não	13,6	12,0-15,2	1,69	0,96-2,97	0,067	5,51	1,34-22,64	0,018
Sim	8,4	4,7-12,1	1,00	-		1,00	-	

IC95%: intervalo de 95% de confiança.

Fonte: Groisman et al. ^22^.

* Ajustada pela idade.

O tempo de cuidado da pessoa idosa, raça/cor e aumento acentuado do tempo de dedicação ao cuidar na pandemia não apresentaram associação significativa com o apoio contratado em nenhum dos modelos analisados. Outras variáveis perderam associação no modelo de análise multivariado, incluindo a situação de pobreza, situação de trabalho antes da pandemia, problema crônico de coluna e estado de ânimo muito ruim (Tabela 2).

De acordo com o modelo de análise multivariado (Tabela 2), a prevalência de apoio contratado é 5,51 vezes maior (IC95%: 1,34-22,64) entre aquelas que não relataram uma diminuição acentuada na renda familiar do que entre as que relataram. Outros fatores associados incluem: não dedicar-se ao cuidado em tempo integral (RPajustada = 3,02; IC95%: 1,62-5,64); autoavaliar a própria saúde como boa/excelente (RPajustada = 2,83; IC95%: 1,58-5,05); cuidar de idoso com alta dependência (RPajustada = 2,14; IC95%: 1,47-3,13); não residir com a pessoa idosa cuidada (RPajustada = 1,95; IC95%: 1,31-2,90); relatar um aumento acentuado no esforço necessário ao cuidado na pandemia (RPajustada = 1,86; IC95%: 1,06-3,26); e não realizar atividades domésticas como parte do cuidado (RPajustada = 1,80; IC95%: 1,24-2,59). Morar em domicílios com um a dois moradores foi negativamente associado à presença do suporte contratado na análise multivariada (RPajustada = 0,68; IC95%: 0,49-0,95).

A prevalência de apoio familiar entre as cuidadoras familiares de pessoas idosas foi de 48,6% (IC95%: 46,4-50,8), ultrapassando os 60% aquelas que não residiam com a pessoa idosa cuidada e que não realizavam atividades domésticas entre as tarefas do cuidado (Tabela 3). A menor prevalência foi observada entre as que se dedicavam ao cuidado em tempo integral (35,2%; IC95%: 31,8-38,6).

**Tabela 3:** Prevalência e razões de prevalência (RP), bruta e ajustada, das características socioeconômicas e de saúde relacionadas ao cuidado de cuidadores familiares que recebem apoio familiar. Brasil, 2020 (n = 2.203).

Variáveis	%	IC95%	RP	IC95%	Valor de p	RPajustada *	IC95%	Valor de p
Total	48,6	46,4-50,8	-	-	-	-	-	-
Características socioeconômicas e do cuidado independentes da pandemia								
Raça/Cor da pele								
Branca	45,6	42,5-48,7	1,00	-	0,108	1,00	-	0,954
Negra	53,3	50,1-56,5	1,09	0,98-1,21		1,00	0,89-1,11	
Situação de pobreza monetária								
Não pobre	43,3	39,8-46,8	1,00	-	0,002	1,00	-	0,344
Pobre	53,4	50,3-56,5	1,22	1,08-1,39		1,06	0,93-1,21	
Situação do trabalho antes da pandemia								
Trabalhava	52,6	49,1-56,1	1,13	1,02-1,25	0,019	1,14	1,02-1,26	0,018
Não trabalhava	46,0	43,2-48,8	1,00	-		1,00	-	
Número de moradores no domicílio								
1 ou 2	41,2	36,8-45,6	1,00	-	0,024	1,00	-	< 0,001
3 ou mais	52,0	49,4-54,6	1,17	1,02-1,33		1,38	1,18-1,60	
Alto grau de dependência								
Não	51,6	48,5-54,7	1,15	1,04-1,27	0,008	1,02	0,91-1,13	0,769
Sim	45,6	42,5-48,7	1,00	-		1,00	-	
Tempo que cuida da pessoa idosa (anos)								
Até 3	55,6	52,1-59,1	1,24	1,11-1,38	< 0,001	1,12	1,00-1,25	0,046
Mais de 3	44,2	41,4-47,0	1,00	-		1,00	-	
Condições de saúde, cuidado e renda atreladas à pandemia								
Autoavaliação da saúde								
Boa/Excelente	51,2	48,3-54,1	1,09	0,98-1,21	0,106	0,91	0,82-1,03	0,129
Moderada/Ruim/Péssima	46,2	42,8-49,6	1,00	-		1,00	-	
Problema crônico de coluna								
Não	50,8	47,8-53,8	1,10	0,99-1,22	0,071	0,98	0,87-1,09	0,681
Sim	45,7	42,5-48,9	1,00	-		1,00	-	
Estado de ânimo muito ruim								
Não	52,0	49,1-54,9	1,20	1,08-1,33	0,001	1,01	0,90-1,13	0,906
Sim	44,3	41,0-47,6	1,00	-		1,00	-	
Dedicação ao cuidado em tempo integral								
Não	56,6	53,8-59,4	1,66	1,46-1,87	< 0,001	1,46	1,26-1,69	< 0,001
Sim	35,2	31,8-38,6	1,00	-		1,00	-	
Reside com a pessoa idosa								
Não	61,7	58,1-65,3	1,48	1,34-1,63	< 0,001	1,36	1,21-1,53	< 0,001
Sim	41,7	39,0-44,4	1,00	-		1,00	-	
Realiza atividades domésticas entre as tarefas do cuidado								
Não	60,7	55,2-66,2	1,40	1,25-1,57	< 0,001	0,96	0,83-1,11	0,604
Sim	46,5	44,1-48,9	1,00	-		1,00	-	
Aumento acentuado do tempo dedicado ao cuidado								
Não	52,1	49,2-55,0	1,24	1,11-1,38	< 0,001	0,88	0,77-1,02	0,087
Sim	43,8	40,5-47,1	1,00	-		1,00	-	
Aumento acentuado do esforço dedicado ao cuidado								
Não	52,8	50,0-55,6	1,31	1,17-1,47	< 0,001	1,51	1,28-1,77	< 0,001
Sim	42,0	38,5-45,5	1,00	-		1,00	-	
Diminuição acentuada da renda familiar								
Não	47,4	45,1-49,7	1,00	-	0,002	1,00	-	< 0,001
Sim	58,6	52,0-65,2	1,25	1,08-1,43		1,42	1,24-1,63	

IC95%: intervalo de 95% de confiança.

Fonte: Groisman et al. ^22^.

* Ajustada pela idade.

A raça/cor, autoavaliação da saúde e problema crônico de coluna não apresentaram associação com o apoio familiar em nenhum dos modelos analisados (Tabela 3). Algumas variáveis perderam significância após o ajuste, incluindo a situação de pobreza, o alto grau de dependência da pessoa idosa cuidada, a realização de atividades domésticas entre as tarefas do cuidado e o aumento acentuado no tempo de dedicação ao cuidado na pandemia.

O modelo ajustado demonstra que a prevalência de apoio familiar é maior entre aquelas que não relataram um aumento acentuado do esforço necessário ao cuidado na pandemia (RPajustada = 1,51; IC95%: 1,28-1,77), não se dedicavam ao cuidado em tempo integral (RPajustada = 1,46; IC95%: 1,26-1,69) e que tiveram a renda muito diminuída na pandemia (RPajustada = 1,42; IC95%: 1,24-1,63). Ter trabalho remunerado antes da pandemia, morar em domicílios com três ou mais habitantes, cuidar da pessoa idosa há menos de três anos e não residir com ela foram características que também apresentaram associação, porém com menor intensidade (RPajustada < 1,40).

A prevalência de cuidadoras familiares com ausência de apoio é de 38,3% (IC95%: 36,2-40,4) (Tabela 4). Entre cuidadoras que se dedicavam ao cuidado em tempo integral, esse percentual alcançava 60%. Em contraste, menos de 20% das que não realizavam atividades domésticas entre as tarefas do cuidado e que não residiam com a pessoa idosa cuidada estavam desassistidas.

**Tabela 4:** Prevalência e razões de prevalência (RP), bruta e ajustada, das características socioeconômicas e de saúde relacionadas ao cuidado de cuidadores familiares que tem ausência de apoio. Brasil, 2020 (n = 2.203).

Variáveis	%	IC95%	RP	IC95%	Valor de p	Rpajustada *	IC95%	Valor de p
Total	38,3	36,2-40,4	-	-	-	-	-	-
Características socioeconômicas e do cuidado independentes da pandemia								
Raça/Cor da pele								
Branca	40,2	37,2-43,2	1,07	0,94-1,22	0,304	0,96	0,84-1,10	0,544
Negra	36,2	33,1-39,3	1,00	-		1,00	-	
Situação de pobreza monetária								
Não pobre	40,4	36,9-43,9	1,11	0,96-1,28	0,160	1,10	0,94-1,28	0,223
Pobre	36,9	33,9-39,9	1,00	-		1,00	-	
Situação do trabalho antes da pandemia								
Trabalhava	30,5	27,3-33,7	1,00	-	< 0,001	1,00	-	0,003
Não trabalhava	43,4	40,6-46,2	1,46	1,26-1,69		1,26	1,08-1,47	
Número de moradores no domicílio								
1 ou 2	43,1	38,7-47,5	1,11	0,96-1,28	0,159	1,55	1,35-1,79	< 0,001
3 ou mais	36,8	34,3-39,3	1,00	-		1,00	-	
Alto grau de dependência								
Não	39,3	36,3-42,3	1,04	0,91-1,17	0,585	1,22	1,07-1,41	0,004
Sim	37,3	34,3-40,3	1,00	-		1,00	-	
Tempo que cuida da pessoa idosa (anos)								
Até 3	30,6	27,3-33,9	1,00	-	< 0,001	1,00	-	0,073
Mais de 3	43,1	40,4-45,8	1,45	1,25-1,67		1,17	0,99-1,38	
Condições de saúde, cuidado e renda atreladas à pandemia								
Autoavaliação da saúde								
Boa/Excelente	32,3	29,6-35,0	1,00	-	< 0,001	1,00	-	0,030
Moderada/Ruim/Péssima	45,7	42,4-49,0	1,38	1,22-1,57		1,18	1,02-1,36	
Problema crônico de coluna								
Não	34,7	31,8-37,6	1,00	-	< 0,001	1,00	-	0,710
Sim	42,6	39,5-45,7	1,25	1,11-1,42		1,03	0,90-1,18	
Estado de ânimo muito ruim								
Não	33,3	30,6-36,0	1,00	-	< 0,001	1,00	-	0,561
Sim	44,8	41,5-48,1	1,39	1,23-1,57		0,96	0,83-1,11	
Dedicação ao cuidado em tempo integral								
Não	24,8	22,4-27,2	1,00	-	< 0,001	1,00	-	< 0,001
Sim	60,6	57,1-64,1	2,53	2,22-2,88		1,62	1,39-1,89	
Reside com a pessoa idosa								
Não	16,1	13,4-18,8	1,00	-	< 0,001	1,00	-	< 0,001
Sim	49,9	47,2-52,6	3,28	2,69-3,99		2,76	2,12-3,60	
Realiza atividades domésticas entre as tarefas do cuidado								
Não	17,8	13,5-22,1	1,00	-	< 0,001	1,00	-	0,064
Sim	41,9	39,6-44,2	2,71	1,99-3,70		1,34	0,98-1,84	
Aumento acentuado do tempo dedicado ao cuidado								
Não	35,7	32,9-38,5	1,00	-	0,002	1,00	-	0,029
Sim	41,9	38,6-45,2	1,22	1,08-1,38		0,82	0,69-0,98	
Aumento acentuado do esforço dedicado ao cuidado								
Não	34,7	32,1-37,3	1,00	-	< 0,001	1,00	-	< 0,001
Sim	44,1	40,6-47,6	1,33	1,17-1,50		1,45	1,22-1,73	
Diminuição acentuada da renda familiar								
Não	38,9	36,6-41,2	1,20	0,95-1,51	0,118	1,26	1,01-1,57	0,044
Sim	33,0	26,7-39,3	1,00	-		1,00		

IC95%: intervalo de 95% de confiança.

Fonte: Groisman et al. ^22^.

* Ajustada pela idade.

Raça/cor e situação de pobreza não apresentaram associação significativa com a ausência de apoio ao cuidado em nenhum dos modelos estimados (Tabela 4). Problema crônico de coluna, estado de ânimo muito ruim e realização de atividades domésticas entre as tarefas do cuidado foram características que perderam associação quando ajustadas pelos demais fatores. Por outro lado, fatores não associados no modelo bivariado demonstraram-se associados após o ajuste do modelo: residir em domicílio com uma ou duas pessoas (RPajustada = 1,55; IC95%: 1,35-1,79); não ter a renda muito diminuída na pandemia (RPajustada = 1,26; IC95%: 1,01-1,57); e o cuidado de pessoa idosa sem alto grau de dependência (RPajustada = 1,22; IC95%: 1,07-1,41). Por fim, o aumento acentuado do tempo de dedicação ao cuidado na pandemia inverteu o sentido de sua associação após o ajuste do modelo (RPajustada = 0,82; IC95%: 0,69-0,98).

Outras associações com a ausência de apoio identificadas no modelo múltiplo (Tabela 4) foram verificadas com os seguintes fatores: residir com a pessoa idosa (RPajustada = 2,76; IC95%: 2,12-3,60); a dedicação ao cuidado em tempo integral (RPajustada = 1,62; IC95%: 1,39-1,89); o aumento acentuado do esforço dedicado ao cuidado na pandemia (RPajustada = 1,45; IC95%: 1,22-1,73); não trabalhar de forma remunerada antes da pandemia (RPajustada = 1,26; IC95%: 1,08-1,47); e a autoavaliação da saúde moderada/ruim/péssima (RPajustada = 1,18; IC95%: 1,02-1,36).

## Discussão

Nesta pesquisa, verificou-se que a divisão da responsabilidade do cuidado de pessoas idosas é determinante para evitar cargas elevadas e para a preservação da saúde, corroborando evidências de estudos anteriores ^8^^,^^10^^,^^11^. Demonstrou-se também que o apoio familiar é mais comum entre as cuidadoras mais pobres e mais impactadas economicamente pela pandemia. Cuidadoras que contavam com apoio contratado, por sua vez, estavam menos expostas à sobrecarga de trabalho e conseguiram, com maior frequência, preservar sua saúde e renda no período.

A prevalência de cuidadoras familiares sem apoio foi de 38,3%, superior ao observado em outro estudo realizado para municípios de diferentes regiões do Brasil no ano de 2019 (28,6%) ^7^, possivelmente devido à pandemia. Sendo a idade e a presença de comorbidades (característica comum entre pessoas idosas que dependem de cuidados ^26^) fatores agravantes da COVID-19 e de seu potencial de letalidade ^27^, é concebível que os cuidadores familiares de pessoas idosas tenham seguido as medidas preventivas com maior rigor, reduzindo o número de pessoas em contato com a pessoa cuidada, incluindo ajudantes ^28^^,^^29^.

O estado de ânimo ruim foi menos frequente entre cuidadoras com apoio, contratado ou não. Estudos anteriores ao período pandêmico apresentaram resultados semelhantes, especialmente em relação ao mal-estar emocional, sintomas depressivos e/ou de ansiedade em cuidadores familiares sem apoio ^8^^,^^10^^,^^11^.

A maioria (56%) das cuidadoras familiares estava em situação de pobreza monetária, o que é superior ao nível nacional estimado pela PNAD em 2020 (29%) ^24^. O ato de cuidar exige tempo e dinheiro, e pode resultar em perdas de oportunidades no mercado de trabalho. Na ausência de políticas que assegurem uma renda adequada, muitas das cuidadoras familiares ficam sujeitas à escassez de recursos materiais básicos ^30^. Camarano ^31^ alertou que essa situação iria piorar na pandemia.

As cuidadoras com apoio contratado geralmente têm melhores condições socioeconômicas, resultado que está em consonância com estudos prévios ^21^^,^^32^. Famílias de baixa renda dificilmente têm recursos suficientes para custear um cuidador contratado. Nesse sentido, políticas públicas que forneçam serviços formais de cuidado domiciliar ^31^ poderiam atenuar essa realidade.

O cuidado em tempo integral e a realização de atividades domésticas foi menos relatado pelas cuidadoras que contavam com apoio contratado. Um estudo nos Estados Unidos analisou o apoio emocional e prático fornecido às cuidadoras familiares e descobriu que, embora o apoio da família esteja associado a um maior suporte emocional, os provedores de apoio contratados oferecem mais assistência prática para as atividades de cuidado ^33^. Anjos ^8^, por sua vez, aponta que o caráter informal do apoio familiar geralmente ocorre com ações pontuais nos momentos de emergências e necessidades, sendo, no geral, insuficiente para a distribuição efetiva da prestação de cuidados.

O alto grau de dependência da pessoa idosa demonstrou-se fortemente associado com a presença do apoio contratado. Miller & McFall ^34^, ao observarem situação similar em um estudo longitudinal, verificaram que o aumento da sobrecarga dos cuidadores familiares, decorrente da piora da saúde e funcionalidade da pessoa idosa cuidada, é o principal motivo que os leva a buscar esse tipo de suporte.

A pior situação socioeconômica das cuidadoras que recebem apoio familiar pode ser explicada de duas maneiras. Em famílias com poucos recursos, o suporte familiar é a única opção para aliviar o impacto do cuidado de dependentes ^35^. Adicionalmente, as famílias mais pobres geralmente vivem em casas com mais moradores ^36^, o que significa que há mais parentes disponíveis para prestar apoio.

A maioria das variáveis relacionadas à sobrecarga do trabalho, como dedicação ao cuidado em tempo integral, coabitação e atividades domésticas, apontam para as cuidadoras sem apoio, o que está de acordo com a literatura ^8^. Destaca-se, ainda, que as cuidadoras com ausência de apoio também estiveram mais sujeitas a um aumento acentuado no esforço dedicado ao cuidado, o que indica piora da sobrecarga. O aumento do tempo de dedicação ao cuidado na pandemia, por outro lado, foi mais relatado pelas que contam com apoio familiar, sendo a ausência de apoio um fator inversamente associado a esse evento. Uma hipótese possível é a de que cuidadoras sem apoio, em grande parte, já se dedicavam integralmente ao cuidado antes da pandemia, tornando o tempo de dedicação menos suscetível a pioras.

Durante a pandemia de COVID-19, com a adesão ao distanciamento social por muitas famílias ^37^, o tempo de convívio entre aquelas que moram com a pessoa idosa dependente de cuidados aumentou consideravelmente, tornando a carga do cuidado ainda maior. Isso afetou principalmente aquelas que não contavam com nenhum tipo de ajuda, tendo em vista que residir com o idoso foi a característica mais associada a esse grupo.

Cuidadoras sem apoio atuavam há mais tempo do que aquelas com apoio (remunerado ou familiar). Cuidados de longo prazo dificultam a continuidade da rede de suporte ^38^. Isso ocorre porque, com o tempo, membros da família que prestavam apoio deixam de prestar. A estruturação do cuidado no ambiente familiar, junto à tentativa de equilibrar as necessidades dos cuidadores e dos demandantes do cuidado, geram tensões entre os membros da família ^38^, que se agravam à medida que o cuidador principal assume mais responsabilidades e se torna dominante na tomada de decisão, desconsiderando, às vezes, a opinião dos outros membros da família e da própria pessoa idosa ^39^.

Do total de cuidadoras familiares, 44% relataram um estado de ânimo negativo durante a pandemia, muito frequentemente sentindo-se ansiosas/nervosas e tristes/deprimidas. Um estudo pré-pandemia realizado com cuidadores familiares chineses constatou que 43,1% deles relataram sintomas de ansiedade. Embora os conceitos adotados e os períodos analisados sejam distintos, é notável a similaridade entre as prevalências observadas nas duas pesquisas. Em contrapartida, estudo na Espanha encontrou uma prevalência relativamente maior (65%) de cuidadores com sintomas de ansiedade ^40^.

Estudos internacionais ^41^^,^^42^ e nacionais ^29^^,^^31^ relataram que a pandemia de COVID-19 agravou a precária situação de vida dos cuidadores familiares. Esse quadro é explicado por fatores como: o encarecimento de recursos necessários para o cuidado ^43^, associado à fragilização da situação econômica dos cuidadores no cenário de crise econômica ^44^; o medo de contaminar a pessoa idosa cuidada, devido à maior letalidade entre elas ^45^; e o distanciamento social necessário para a redução da disseminação do vírus, que aumentou o sentimento de solidão entre os cuidadores ^41^^,^^42^ e exigiu o afastamento de ajudantes externos ao lar ^29^.

Dos cuidadores familiares entrevistados no inquérito CUIDA-COVID, 92% eram do sexo feminino ^22^, percentual próximo ao identificado em estudo realizado em um Município de São Paulo, segundo o qual 85,7% dos cuidadores informais eram mulheres ^46^. A construção social da feminização do cuidado é derivada da histórica divisão sexual do trabalho, na qual os homens se destinam às ocupações da esfera pública, da vida econômica e política, enquanto as mulheres se ocupam da esfera privada, da domesticidade e reprodução, em atividades subvalorizadas ^17^.

No Brasil, a história da prática do cuidar guarda questões raciais de igual importância ^16^^,^^47^. Durante o período colonial e imperial, mulheres negras foram forçadas a trabalhar em atividades de cuidado como amas de leite, babás, pretas-velhas e parteiras ^16^. A abolição da escravatura não modificou as estruturas hierárquicas da lógica escravista e, em grande medida, elas continuaram exercendo as mesmas funções, agora sob a lógica do mercado ^48^. Assim, a formação sócio-histórica brasileira e o racismo estrutural dela decorrente geraram a “terceirização do trabalho doméstico”, na qual mulheres negras, além de se encarregarem do cuidado de suas próprias famílias, atuam no cuidado de famílias brancas ^49^.

Neste trabalho, entretanto, verificou-se que a presença e o tipo de apoio pouco variaram entre cuidadoras negras e brancas, o que contrasta com estudo estadunidense que constatou que negras tinham probabilidade 50% maior de serem as únicas responsáveis pelo cuidado do que brancas ^33^.

No Brasil, as poucas práticas e ações estatais com foco no suporte ao cuidado de pessoas dependentes são localizadas ^3^. Uma das formas de atenção estatal aos cuidadores, em nível nacional, é ofertada por meio de ações da Estratégia Saúde da Família (ESF). No entanto, tais medidas geralmente se direcionam à pessoa idosa demandante de cuidados ^3^. O fortalecimento da integração familiar com a ESF, de modo a ampliar a oferta de assistência e acompanhamento integral também aos cuidadores familiares, é uma medida relativamente simples e absolutamente fundamental ^50^. No entanto, a assistência à saúde não basta. Fornecer apoio via cuidadores formais, prover recursos financeiros e materiais e disponibilizar orientações sobre o manejo das doenças/condições incapacitantes da pessoa idosa são medidas que, adequadamente combinadas, serão efetivas para atender as necessidades de saúde e de vida dessa população ^31^.

Duas limitações do estudo devem ser apontadas. Por ser um questionário virtual, a pesquisa tende a abranger uma população com maior escolaridade e inclusão digital. Com isso, é possível que a prevalência do apoio familiar esteja subestimada. Por último, destaca-se que a variável do apoio ao cuidado tornou possível identificar o tipo ou a ausência do apoio recebido, no entanto, sua intensidade, que provavelmente apresenta uma relação causal mais adequada com as condições de vida das cuidadoras, não foi mensurada pelo estudo.
